# Argonaute 2 sustains the gene expression program driving human monocytic differentiation of acute myeloid leukemia cells

**DOI:** 10.1038/cddis.2013.452

**Published:** 2013-11-21

**Authors:** I Iosue, R Quaranta, S Masciarelli, G Fontemaggi, E M Batassa, C Bertolami, T Ottone, M Divona, B Salvatori, F Padula, A Fatica, F Lo-Coco, C Nervi, F Fazi

**Affiliations:** 1 Department of Medico-Surgical Sciences and Biotechnologies, Sapienza University of Rome, Latina 04100, Italy; 2 San Raffaele Bio-Medical Park Foundation, Rome 00128, Italy; 3 Translational Oncogenomics Unit, ‘Regina Elena' National Cancer Institute, Rome 00144, Italy; 4 Department of Biopathology, University of Rome Tor Vergata, Rome 00133, Italy; 5 Laboratory of Neuro-Oncoematology, Santa Lucia Foundation, Rome 00143, Italy; 6 Department of Biology and Biotechnology ‘Charles Darwin' and Institute Pasteur Cenci-Bolognetti, Sapienza University of Rome, Rome, Italy; 7 Section of Histology and Medical Embriology, DAHFMO, University of Rome ‘La Sapienza', Rome 00161, Italy

**Keywords:** microRNA, argonaute 2, acute myeloid leukemia, myeloid differentiation

## Abstract

MicroRNAs are key regulators of many biological processes, including cell differentiation. These small RNAs exert their function assembled in the RNA-induced silencing complexes (RISCs), where members of Argonaute (Ago) family of proteins provide a unique platform for target recognition and gene silencing. Here, by using myeloid cell lines and primary blasts, we show that Ago2 has a key role in human monocytic cell fate determination and in LPS-induced inflammatory response of 1,25-dihydroxyvitamin D_3_ (D3)-treated myeloid cells. The silencing of Ago2 impairs the D3-dependent miR-17-5p/20a/106a, miR-125b and miR-155 downregulation, the accumulation of their translational targets AML1, VDR and C/EBP*β* and monocytic cell differentiation. Moreover, we show that Ago2 is recruited on miR-155 host gene promoter and on the upstream region of an overlapping antisense lncRNA, determining their epigenetic silencing, and miR-155 downregulation. These findings highlight Ago2 as a new factor in myeloid cell fate determination in acute myeloid leukemia cells.

Normal hematopoietic progenitors' development, proliferation and lineage differentiation are largely controlled by a unique combination of lineage-specific transcription factors, which cooperatively regulate the activity of promoters and enhancers present on their target genes.^1^ Recent findings, however, indicate that microRNAs (miRNAs) also affect hematopoiesis at different stages. MiRNAs have been found present in integrated regulatory feedback loops in which they can change lineage transcription factors levels or can themselves be regulated by these factors. These integrated circuitries involving transcription factors and miRNAs lead to appropriate decisions regarding proliferation and differentiation status in different cell types, including hematopoietic cells.^2, 3^

Thus, modulation of specific transcription factors and miRNA levels or functions affects self-renewal capacity, proliferation and differentiation of myeloid progenitors resulting in myeloproliferative disorders and acute myeloid leukemias (AMLs).^4^

Leukemic cells exhibit a maturation block at specific hematopoietic differentiation stages that characterizes the different leukemia subtypes, classified as AML-M0 to AML-M7 according to FAB classification.^5^ Of note, the maturation block underlying specific AML subtypes can be overcome by treatment with physiologic inducers such as the retinoic acid (RA) or 1,25-dihydroxyvitamin D_3_ (D3).^6^ RA and D3 are able to trigger the human myeloid precursors to differentiate into either granulocytes or monocytes, respectively.^7^

To perform their regulatory functions, miRNAs must assemble with any of the four mammalian Argonaute (Ago) family of proteins, Ago1–4, into an effector complex known as the RNA-induced silencing complex (RISC).^8, 9, 10^ Ago proteins are characterized by a conserved structure consisting of an amino-terminal domain, a so-called Mid (middle) domain, the PAZ (PIWI-Argonaute-Zwille) and Piwi (P-element-induced wimpy testes) domains. Whereas the Piwi domain structurally resembles an RNase H fold, the PAZ and Mid domains bind to the 3′- and 5′-end of the miRNAs, respectively.^11, 12^ The miRNAs loaded in the RISC complex usually function as negative regulators of gene expression, mainly by targeting the 3′ untranslated region (UTR) of their target mRNAs.^13, 14^ In general, while the mature miRNA guides the RISC complex to its target mRNA, the Ago protein complex represses mRNA translation or induces deadenylation-dependent mRNA decay, leading to silencing of gene expression.^15, 16, 17, 18^ Of note, despite a remarkable homology that extends to the PIWI domains among all four human Ago proteins, Argonaute2 (Ago2) is the only human Ago protein endowed with nuclease activity.^19^ In this context, a novel role for the Ago2 slicer catalytic activity was recently identified in the Dicer-independent miRNA precursor maturation.^20^ Moreover, Argonaute–RNA complexes can regulate nuclear events like transcription, genome maintenance and splicing.^21^ Recently, both Ago1 and Ago2 were found in the nucleus of mammalian cells.^22^ In human cells, they present specific nuclear compartment localization and contribute to transcriptional gene silencing (TGS).^23, 24, 25^ Ago1 and Ago2 were found associated with promoter gene sequences, and the inhibition of their expression reverses transcriptional and post-transcriptional silencing.^24, 26^ In particular, it was recently evidenced that transcriptional silencing mediated by single-stranded RNAs complementary to an antisense long noncoding RNA (lncRNA) overlapping progesterone receptor promoter requires the presence of Ago2 protein.^27, 28, 29^ Moreover, emerging evidence also indicates that Ago2 is necessary to maintain a constitutive heterochromatin genomic structure at beta-globin locus in vertebrate. The reduction of Ago2 expression or the treatment with an HDAC inhibitor, such as the trichostatin A (TSA), mainly increases the acetylation status of histone H4 and induces the chromatin to adopt a more open structure.^30^ Although growing evidence links the epigenetic status of miRNAs regulatory regions to the modulation of their expression levels in humans, the possible involvement of Ago proteins in the epigenetic regulation of miRNAs transcription still remains elusive.

Gene inactivation experiments showed that Ago2 protein is crucial for embryonic development, stem cell maintenance and cell differentiation.^31, 32^ In addition, recent evidence identifies Ago2 as a key regulator of B lymphoid and erythroid development and function.^33^ In Ago2^−/−^ mice, the loss of Ago2-mediated translational control impairs miRNAs biogenesis. The reduced threshold of miRNA-mediated gene silencing results in hematopoietic defects, including erythroid hyperplasia, splenomegaly and severe anemia.^33^ Moreover, recently, a crucial role for Ago proteins in maintaining mature miRNA homeostasis is also emerging in T cells. Ago2-deficient T cells display reduced miRNAs levels and a greater capability to differentiate into cytokine-producing effectors.^34^

In this study, we addressed the involvement of Ago2 in the regulation of human myeloid differentiation and leukemogenesis. We observed that Ago2 protein levels are increased during monocyte differentiation of myeloid progenitors, whereas Ago2 is downregulated during granulocyte differentiation of human leukemic cell lines and freshly isolated blasts from acute promyelocytic leukemia patients. We found that Ago2 depletion alters the correct modulation of transcription factors and microRNAs involved in monocyte cell fate determination, leading to the reduction of D3-induced monocytic differentiation and activation.

## Results

### Ago2 protein is modulated during human myeloid lineage determination

**Figure 1:**
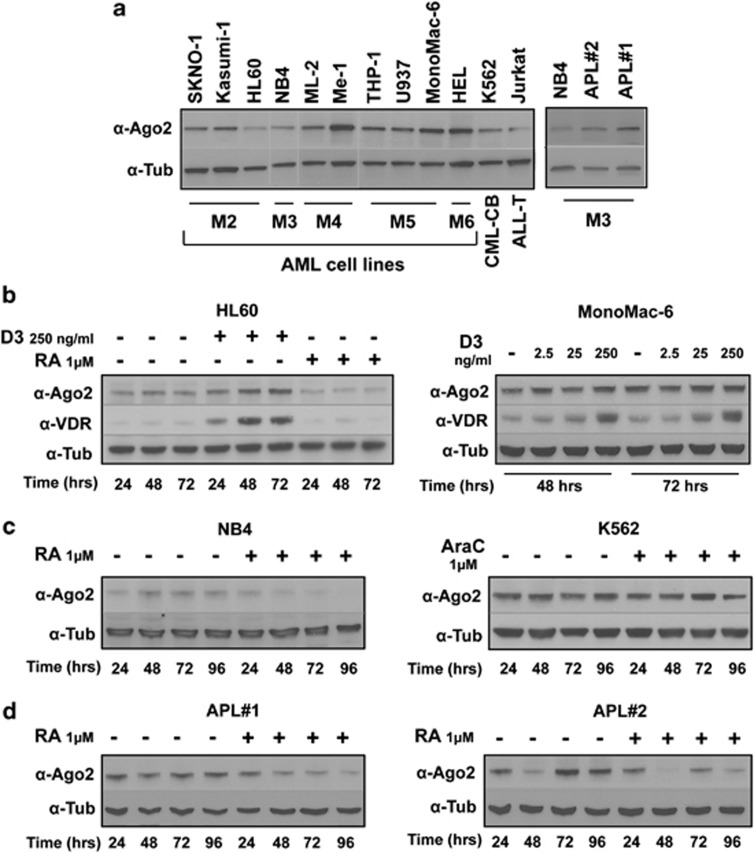
Ago2 protein levels in human AML cell lines and during myeloid differentiation. (**a**) Immunoblot analysis for the detection of Ago2 in the indicated AML cell lines classified by FAB (AML-M0 to AML-M7) according to their morphologic and cytochemical characteristics^5^ and in primary blasts from BM (APL#1) and PB (APL#2) of two newly diagnosed APL cases. CML-BC and ALL are chronic myelogenous leukemia in blast crisis and acute lymphocytic leukemia cell lines, respectively. (**b**) Immunoblot analysis for the detection of vitamin D receptor (VDR) and Ago2 in the myeloblastic HL-60 cell line treated or not with 1 *μ*M of retinoic acid (RA) or with 250 ng/ml of 1,25-dihydroxyvitamin D_3_ (D3) at the indicated times (left panels) or in the monocytic MonoMac-6 cell line after treatment for 48 or 72 h with increasing doses of D3 (2.5–25–250 ng/ml) (right panels). (**c** and **d**) Immunoblot analysis for the detection of Ago2 in NB4 (APL-M3) and CML-BC K562 cell lines treated or not with 1 *μ*M of RA or ARA-C as indicated and in primary blasts described in (A) prior or after *in vitro* treatment with RA (1 *μ*M). The levels of tubulin visualized an equal amount of protein loading. Densitometric analysis is reported in Supplementary Figure 1

By analyzing the expression levels of the Argonaute family member Ago2 in a panel of human hematopoietic cell lines, we observed that Ago2 shows the lowest levels of expression in 2 out of 12 cell lines, that is, the HL60 (AML-M2) and NB4 (AML-M3), exhibiting a myeloblastic and promyelocytic phenotype, respectively^35, 36^ (Figure 1a; Supplementary Figure S1). HL60 and NB4 cells are distinguished by their marked sensibility to differentiation treatments and both can be triggered to terminally differentiate into granulocytes upon exposure to retinoic acid (RA),^37^ whereas HL60 can also differentiate in monocytes when treated with 1,25-dihydroxyvitamin D_3_ (D3). When Ago2 protein levels were analyzed in HL60 and NB4 cell lines during the *in vitro* treatment with pharmacological doses of RA (1 *μ*M), a reduction in the expression levels of Ago2 mRNA and protein was observed (Figures 1b and c left; Supplementary Figures S1 and S2A and SC). Similar results were obtained with freshly isolated AML-M3 acute promyelocytic leukemia (APL) primary blasts showing a higher Ago2 basal expression level compared with NB4 cells (Figure 1a and d right; Supplementary Figure S1). On the contrary, the treatment with D3 (250 ng/ml), which is able to drive into monocytic differentiation the bipotent HL60 cell line, induced the upregulation of the Ago2 protein without a corresponding increase in the mRNA levels, suggesting that post-transcriptional modifications may be implicated in its accumulation^38^ (Figure 1b left; Supplementary Figures S1 and S2A). Ago2 mRNA and protein levels are not affected by increasing doses of D3 in monocytic MonoMac-6 cell line (AML-M5) (Figure 1b right; Supplementary Figures S1 and S2B). However, in both HL60 and MonoMac-6 cells, the functional activation of the D3 pathway was confirmed by the induction of D3 receptor (VDR) (Figure 1b; Supplementary Figure S1). No changes in Ago2 protein levels occurred in the erythroleukemic K562 cell line after ARA-C-induced erythroid differentiation (Figure 1c right; Supplementary Figure S1). Overall, these results suggested a specific association of Ago2 expression levels and granulocytic/monocytic differentiation program of myeloid progenitors.

### Ago2 expression supports monocytic differentiation of AML cell lines

**Figure 2:**
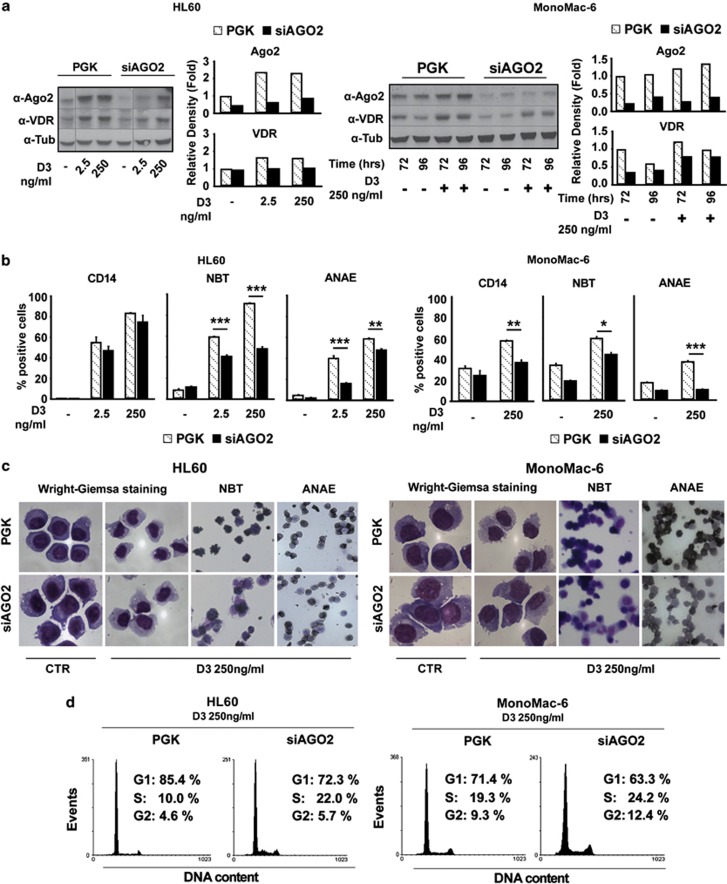
Ago2 depletion decreases the differentiation response to 1,25-dihydroxyvitamin D_3_ of the human myeloblastic (HL60) or monocytic (MonoMac-M6) cell lines. HL60 (*left*) and MonoMac-6 (*right*) cells were infected with an empty lentiviral vector (PGK) or with one expressing shRNAs targeting Ago2 (siAgo2). (**a**) Ago-2 and VDR protein expression levels were analyzed by western blot performed after 96 h of D3 (2.5–250 ng/ml) treatment on total cell lysates (30 *μ*g) from HL60 and after 72 and 96 h of D3 treatment (250 ng/ml) on total cell lysates (30 *μ*g) from MonoMac-6. The graphs on the right of each blot show the respective densitometric analysis in which tubulin was used for normalization. (**b**) Differentiation of HL60 (*left*) and MonoMac-6 treated or not with the indicated doses of D3 was evaluated by the expression of the monocytic marker CD14, using the NBT dye reduction assay and by cytochemical staining for the *α*-naphthyl acetate esterase (ANAE) activity. CD14 expression was assessed by flow cytometry 72 h after the treatments, whereas the NBT reduction and the ANAE activity after 96 h (*n*=3±S.E.M.). Statistical analysis was performed by the Student's *t-*Test (*P value <0.05, **P value <0.005, ***P value <0.0005). (**c**) Light microscopy fields showing morphological changes of HL60 (*left*) and MonoMac-6 (*right*) PGK and siAgo2 cells after 96 h of D3 treatment (250 ng/ml) after Wright-Giemsa staining and representative fields of the NBT and ANAE assays analyzed in (**b** and **d**). Cell cycle analysis performed by flow cytometry on HL60 (*left*) and MonoMac-6 (*right*) PGK and siAgo2 cells after 96 h of D3 treatment (250 ng/ml)

To evaluate the contribution of Ago2 to myeloid cell differentiation, HL-60 and MonoMac-6 cells were infected with a lentiviral vector expressing shRNA directed to Ago2 mRNA. By quantitative RT-PCR (qRT-PCR) and western blot analyses, we assessed a decrease of about 50% in Ago2 mRNA (data not shown) and more than 60% in Ago2 protein levels in siAgo2 cells, compared with mock-infected (PGK) cells (Figure 2a). Notably, this reduction is maintained during D3 treatment and parallels an inhibition of the D3 dose- and time-dependent VDR protein accumulation in HL60- and MonoMac-6-siAgo2-infected cells compared with the mock-infected counterparts (Figure 2a). To evaluate whether the reduced VDR protein accumulation after D3 treatment in siAGO2 cells correlated with an impairment of D3-induced differentiation, we performed both functional and molecular assessment of cell differentiation state.

In HL60-PGK cells the D3 treatment increased, in a dose-dependent manner, monocytic cell differentiation as shown by the increased percentage of cells expressing the CD14 marker, positivity for NBT reduction and ANAE staining (Figure 2b left). The latter was also inhibited by NaF, confirming the specificity of the assay (data not shown). By contrast, such effects were reduced in D3-treated HL60 Ago2-depleted cells. Morphological evaluation showed that, with respect to HL60-PGK cells, D3-treated siAgo2 cells exhibited morphologic features of impaired monocytic maturation, including lack of chromatin condensation, decreased nuclear/cytoplasmic ratio and changes in cytosolic basophilia (Figure 2c left). Moreover, HL60 Ago2-depleted cells showed a decreased cell cycle arrest in response to D3, evidenced by a lower percentage of cells in the G0/G1 phases of the cell cycle relatively to HL60-PGK cells (Figure 2d left). Similar results were obtained in monocytic MonoMac-6-siAgo2-infected cells compared with the mock-infected counterpart (Figures 2b–d right panels).

We noted that, in the D3-treated MonoMac-6, the Ago2 depletion results in a more evident reduction of the CD14 expression than in HL60-siAgo D3-treated cells, and we hypothesize this to be due to the different genetic background of the two cell lines. However, the evident reduction observed after Ago2 depletion in NBT and ANAE staining and morphological changes in D3-treated HL60 and Monomac-6 cell lines strongly support an impaired D3-induced differentiation.

**Figure 3:**
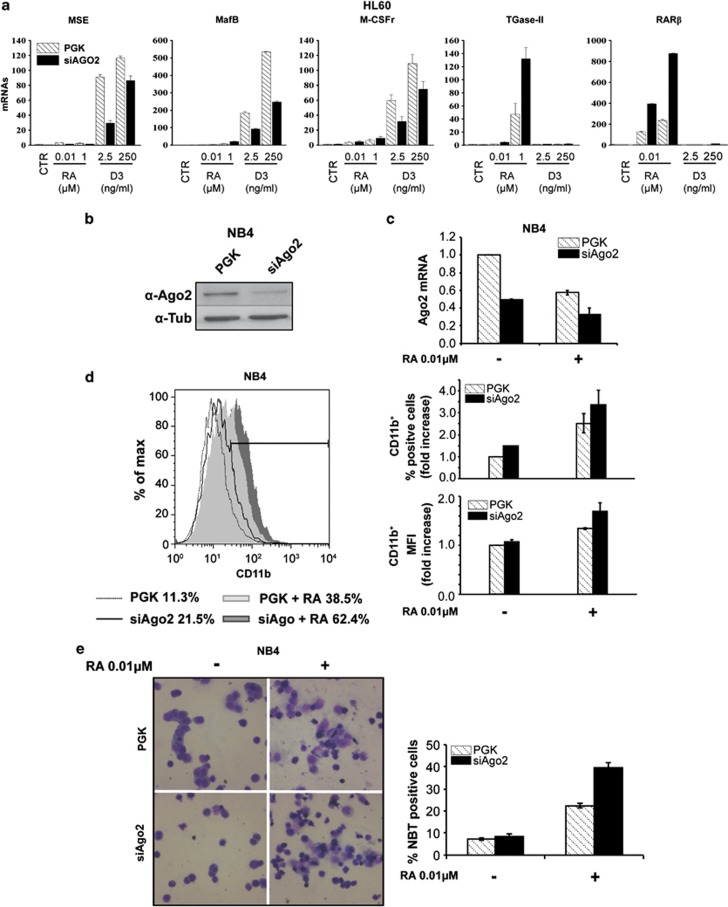
Ago2 silencing promotes RA-induced granulocytic differentiation. (**a**) Dose-dependent expression levels of genes associated with D3-induced monocytic (MSE, MafB and M-CSFr) or RA-induced granulocytic (TGaseII and RAR*β*) differentiation of HL60 PGK or siAgo2 cells evaluated by qRT-PCR analysis. mRNA amounts were evaluated after 72 h of the indicated treatment and normalized with GAPDH values using the ΔΔCt method (*n*=3±S.D.). (**b**) NB4 cells were infected with an empty (PGK) or a shAgo2 lentiviral vector (siAgo2), and the decrease in Ago2 protein levels was measured by western blot. (**c**) The amount of Ago2 mRNA was assessed by qRT-PCR in PGK or siAgo2 cells induced or not to differentiate by RA. Analysis of the data was performed by the ΔΔCt method using GAPDH for normalization of the samples. (**d**) Levels of expression of the surface molecule CD11b during RA-induced granulocytic differentiation of PGK or siAgo2 NB4 cells assessed by flow cytometry. On the left panel an overlay histogram of a representative experiment is shown, whereas on the right panel the graphs report the average fold increase of the percentage of positive cells and of the mean fluorescence intensity (MFI) (*n*=2±S.E.M.). (**e**) NBT dye reduction assay of PGK or siAgo2 NB4 cells. The pictures show typical fields of untreated (RA-) or treated (RA+) NB4 cells. The graph reports the NBT-positive cell counts evaluated after 72 h of treatment of a representative experiment

Real-time PCR analysis revealed that the D3 dose-dependent upregulation of genes associated with myelomonocytic differentiation, such as the monocyte/macrophage serine esterase-1 (MSE), the master regulator of human monocytopoiesis v-maf muscoloaponeurotic fibrosarcoma oncogene homolog B (MafB) and the colony-stimulating factor 1 receptor (CSF1R or M-CSFr), was inhibited in HL60-siAgo2 relatively to HL60-PGK cells (Figure 3a).

As expected, during RA-dependent granulocytic differentiation, we did not observe the induction of the above-mentioned genes (Figure 3a). However, when compared with the mock-infected counterpart, RA-treated HL60-siAgo2 cells showed a strong dose-dependent induction of two RA target genes related to granulocytic differentiation, such as the retinoic-acid-receptor beta (RAR*β*) and the transglutaminase type-II (TGase-II) (Figure 3a).^37^ This observation and the fact that Ago2 expression decreases during RA-induced differentiation (Figures 1b–d) led us to hypothesize a role for Ago2 silencing in granulocyte differentiation of myeloid progenitors and APL cells. We therefore analyzed RA-induced differentiation of NB4 cells, a well-established APL model of granulocytic differentiation, in which Ago2 mRNA and protein were silenced (Figures 3b and c). Upon RA treatment, NB4-siAgo2 cells showed higher percentage and expression levels of CD11b-positive cells (Figure 3d) and an increased capability to reduce the NBT than NB4-PGK (Figure 3e). This indicated a more efficient granulocytic differentiation response to RA treatment of NB4-siAgo2 cells compared with control cells.

Altogether these observations further supported a functional involvement of Ago2 in myeloid cell differentiation.

### Ago2 sustains microRNA/transcription factor regulatory networks related to monocytic cell differentiation

**Figure 4:**
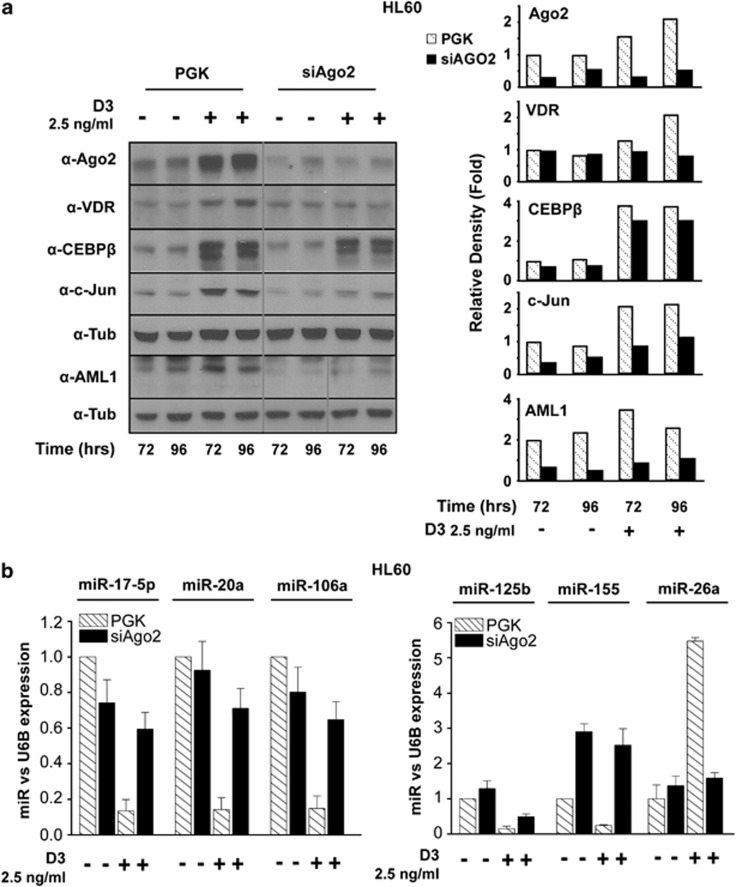
Transcription factors and miRNAs expression during D3-dependent monocyte differentiation. (**a**) Western blot analysis for the detection of the indicated proteins was performed in HL60 PGK and siAgo2 cells after 72 and 96 h of D3 treatment (2.5 ng/ml). The graphs on the right show the densitometric analysis of the blots in which tubulin was used for normalization. Detection of AML-1 was performed on a different nitrocellulose filter relatively to the other proteins, and thus tubulin detected on this filters is shown (bottom row). (**b**) After 72 h of D3 treatment, 10 ng of total RNA from HL60 PGK and siAgo2 cells was retrotranscribed specifically for each indicated miRNAs, and the relative quantification of their expression was measured by qRT-PCR using the ΔΔCt method. Values were normalized to RNU6B as a housekeeping gene (*n*=3±S.D.)

Monocytopoiesis is largely controlled by a unique combination of lineage-specific transcription factors, including vitamin D receptor (VDR), acute myeloid leukemia-1 (AML1) and CCAAT/enhanced-binding protein *β* (C/EBP*β*), which cooperatively regulate promoters or enhancers present on their target gene.^7^ We evaluated whether the impaired D3-induced monocytic differentiation in the absence of Ago2 resides on the deregulation of the above-mentioned transcription factors. To this end, we analyzed their protein levels in the presence or absence of Ago2 expression. Time-course treatments of HL60 cells were performed using 2.5 ng/ml D3, which efficiently affects the expression levels of Ago2, VDR proteins and differentiation markers (Figures 2a and b). During D3-induced monocytic differentiation of HL60-PGK control cells, Ago2 upregulation parallels the increased expression of VDR, AML-1 and C/EBP*β* proteins (Figure 4a).

We therefore explored the involvement of miRNAs in the regulation of these factors expression levels. Interestingly, D3-induced upregulation of AML1, VDR and C/EBP*β* paralleled the downregulation of miR-17-5p/20a/106a cluster, miR-125b and miR-155 (Figure 4b). All these miRNAs have been related to myeloproliferative disorders and leukemia.^39, 40, 41^ Moreover, the miR-17-5p/20a/106a cluster has been previously shown to repress AML1 translation, while miR-125b represses VDR and miR-155 represses C/EBP*β*.^39, 40, 42, 43^

We found that the D3-dependent downregulation of miR-17-5p/20a/106a, miR-125b and miR-155 is impaired in Ago2-depleted HL60 cells (Figure 4b). Accordingly, lower levels of their targets, AML1, VDR and to a lesser extent C/EBP*β*, are detectable in HL60-siAgo2 D3-treated cells when compared with PGK cells (Figure 4a). The relatively small decrease in C/EBP*β* despite miR-155 high amount could be explained by the fact that this transcription factor is highly regulated at multiple levels, and thus the impact of miR-155 could be overcome by other factors.^44^ These results support a contribution of Ago2 to monocytic cell differentiation. Indeed, other monocytic differentiation-associated factors such as miR-26a^45, 46^ and c-Jun are upregulated by D3 treatment, and their accumulation is reduced in HL60-siAgo2 cells in response to D3 (Figures 4a and b right panel).

These findings showed that Ago2 is required for proper modulation of microRNA and their target levels during monocytic differentiation.

### Ago2 localizes at miR-155 host gene promoter and contributes to its heterochromatic silencing

**Figure 5:**
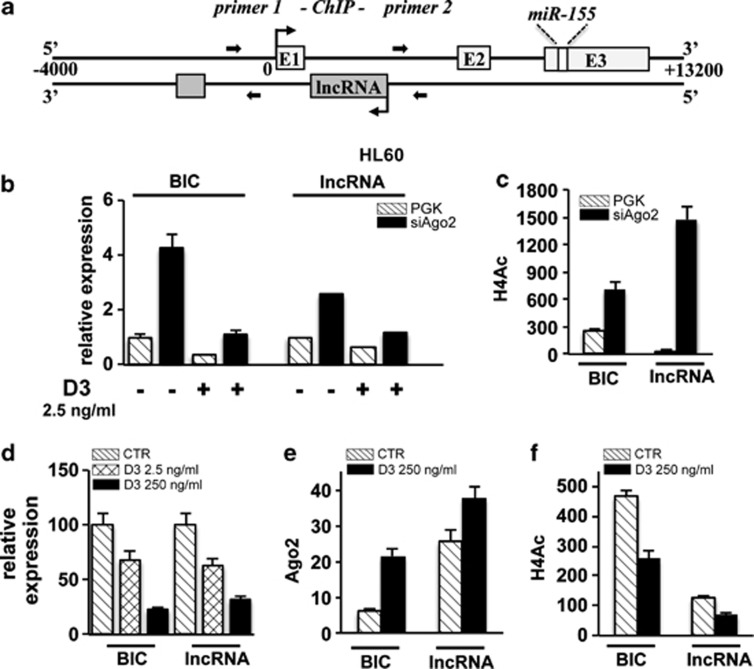
miR-155HG (BIC) and antisense lncRNA-155HG (lncRNA) regulation during monocytic cell differentiation. (**a**) Schematic representation of the genomic structure of human *miR-155* gene as reported by the UCSC Genome Browser website (http://genome.ucsc.edu/). The exon sequences (E) on the *miR-155* gene are numbered (1–3), and the localization of miR-155 mature sequence on E3 is indicated. Arrows indicate the location of the primers used in the ChiP assay, primers 1 for the region upstream miR-155HG and primers 2 for that of lncRNA-155HG. (**b**) After 72 h of D3 treatment total RNA from HL60 PGK and siAgo2 cells was retrotrascribed, and the relative quantification of BIC or lncRNA expression was measured by qRT-PCR using the ΔΔCt method. Values were normalized to RNU6B as a housekeeping gene (*n*=3±S.D.). (**c**) Histograms of the qRT-PCR performed to amplify BIC and lncRNA regions in ChIP assays carried out using an anti-acetyl-Histone-H4 antibody on chromatin samples prepared from HL60-PGK and HL60-siAgo2 cells. Samples were quantified *versus* the respective input and calculated following the 2^−ΔΔCt^ method (*n*=3±S.D.). (**d**) Dose-dependent evaluation of BIC and lncRNA expression associated with D3-induced monocytic differentiation of HL60 cells. RNA amounts were evaluated after 72 h of treatment, and the relative quantification of their expression was measured by qRT-PCR using the ΔΔCt method. Values were normalized to RNU6B as a housekeeping gene (*n*=3±S.D.). (**e** and **f**) Histograms of the qRT-PCR performed to amplify BIC and lncRNA regulatory regions in ChIP assays carried out using an anti-Ago2 antibody (**e**) or an anti-acetyl-Histone-H4 antibody (**f**) on chromatin samples prepared from HL60 cells after 72 h of D3 treatment (250 ng/ml). Samples were quantified *versus* the respective input and calculated following the ΔΔCt method (*n*=3±S.D.)

The up-regulation of miR-155 expression levels detectable in HL60 Ago2-depleted cells (Figure 4b) prompted us to investigate the molecular mechanisms at the basis of the modulation of this miRNA level by Ago2. The analysis of the genomic organization of the miR-155 locus evidenced that the mature sequence of this miRNA is embedded in the third exon of a host gene primary transcript (miR-155HG), also referred to as BIC. In addition, the miR-155HG promoter region overlaps with an antisense long noncoding RNA transcript (referred as TCONS_00028904 in http://www.lncipedia.org) of 2423 bp, which we named lncRNA-155HG (Figure 5a). In untreated HL60-siAgo2 and control cells, we evidenced that the expression of both miR-155HG (BIC) and antisense lncRNA-155HG (Figure 5b) parallels that of miR-155 (Figure 4b). On the contrary, upon D3 treatment we observed a decrease of miR-155HG and lncRNA-155HG in siAgo2 cells that is not reflected in mature miR-155 levels. A similar explanation could reside in the fact that the control of mature miRNAs stability could differ from that of its precursor whose processing efficiency may also vary.^47, 48^ Thus, the amount of transcripts do not necessarily correlate with that of the mature miRNAs. However, it is important to notice that miR-155HG and lncRNA-155HG are still expressed at higher levels in siAgo2 D3-treated cells relatively to the control PGK cells.

Changes in the epigenetic status of miR-155HG promoter have been reported to contribute to the transcriptional control of miR-155 expression levels in B-lymphocytic leukemia (B-CLL) and breast cancer.^49, 50, 51^ We therefore evaluated whether epigenetic modifications occurred on the surrounding miR-155HG and antisense lncRNA-155HG genomic regions following Ago2-depletion by analyzing their chromatin acetylation status. To this end, chromatin immunoprecipitation (ChIP) assay was performed using an antibody recognizing the acetylated form of histone H4 and analyzing the upstream regulatory regions of miR-155HG and antisense lncRNA-155HG. As shown in Figure 5, the upregulation of both miR-155HG and antisense lncRNA-155HG following Ago2 depletion is related to an increase in the acetylation status of histone H4 (Figures 5a–c).

To clarify if Ago2 directly controls miR-155 regulatory regions, we analyzed Ago2 recruitment and histone H4 acetylation status at these sites using ChIP assays in HL60 cells, where both miR-155HG and the antisense lncRNA-155HG show a D3 dose-dependent (2.5-250 ng/ml) downregulation (Figure 5d).

A specific enrichment of miR-155HG and lncRNA-155HG regulatory regions in Ago2-immunoprecipitated samples was observed in HL60 cells (Figure 5e, stripe bars), indicating the binding of Ago2 on these regions. Interestingly, after D3 treatment, we observed an increase in Ago2 recruitment (Figure 5e, black bars) associated with a reduction of histone H4 acetylation status (Figure 5f). Thus, Ago2 recruitment and histone H4 acetylation are inversely correlated on the two analyzed regions in control samples and after D3 treatment (Figures 5e and f).

These findings open to a novel involvement of Ago2 protein in the establishment of a silenced heterochromatin domain at miRNA promoter regions adding a new layer in the epigenetic control of miRNAs biogenesis during cell fate determination processes.

### Ago2 affects the LPS-induced inflammatory response of vitaminD-primed myeloid cells

Recent evidence indicates that miR-155 overexpression inhibits inflammatory cytokine production via targeting C/EBP*β*.^52^ Moreover, miR-155 inhibition was shown to increase LPS-dependent expression of several inflammation mediators in bone marrow-derived macrophages (BMDMs), similarly to the conditional Dicer knockout mice.^53^ The observation that miR-155 expression is increased in Ago2-depleted HL60 (Figure 4b, right panel) prompted us to investigate the role of Ago2 protein in LPS-induced activation.

The granulocyte–macrophage- (GM-CSF) and the macrophage- (M-CSF) colony-stimulating factors are prosurvival/mitogenic factors for monocytes/macrophage lineage populations and contribute to ‘prime' or ‘activate' macrophages as well as to induce their differentiation.^54^ A second stimulus, such as lipopolysaccaride (LPS), is generally required for GM-CSF- or M-CSF-primed monocytes and macrophages to be activated to secrete significant levels of different cytokines.^55^

**Figure 6:**
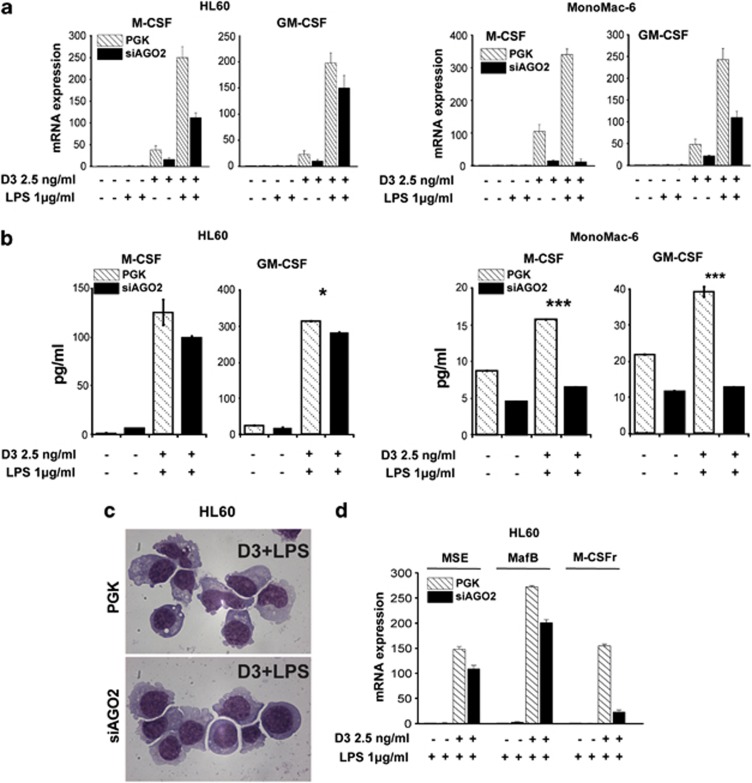
Evaluation of cytokine expression following LPS stimulation in D3-treated human myeloid cells. (**a**) The macrophage (M-CSF) and the granulocyte-macrophage (GM-CSF) colony-stimulating factor mRNA levels evaluated in HL60 (*left*) and MonoMac-6 (*right*) PGK and siAgo2 cells following 72 h of LPS (1 *μ*g/ml) and D3 (2.5 ng/ml) treatment as single agents or combination. The relative expression was measured in qRT-PCR using the ΔΔCt method. Values were normalized to GAPDH as a housekeeping gene (*n*=3±S.D.). (**b**) M-CSF and GM-CSF levels in media collected after 96 h of cultures of HL60 (*left*) and MonoMac-6 (*right*) PGK and siAgo2 cells treated or not with LPS (1 *μ*g/ml) plus D3 (2.5 ng/ml) (*n*=3±S.E.M.). Statistical analysis was performed by the Student's *t-*Test (*P value <0.05, ***P value <0.0005). (**c**) Morphological changes assessed by light-field microscopy of Wright-Giemsa stained HL60 PGK and siAgo2 cells after 96 h of D3 treatment (2.5 ng/ml) plus LPS stimulation (1 *μ*g/ml). (**d**) Expression levels of genes associated with monocytic differentiation (MSE, MafB and M-CSFr) evaluated in HL60 PGK or siAgo2 cells following LPS (1 *μ*g/ml) stimulation as single agents or in combination with D3 treatment (2.5 ng/ml). mRNA amounts were evaluated by qRT-PCR analysis after 72 h of the indicated treatment and normalized with GAPDH using the ΔΔCt method (*n*=3±S.D.)

In this context, in myeloblastic HL60-PGK and monocytic MonoMac-6-PGK mock-infected cell lines, D3 strongly induces M-CSF and GM-CSF mRNA expression and morphological changes related to monocytic differentiation (Figures 6a–c) Interestingly, an additional increase in these cytokine mRNAs is observed in the presence of D3 plus LPS (Figure 6a). LPS alone has no effects on these myeloid precursors in terms of induction of cytokine- and monocytic-related gene expression and morphological changes (Figures 6a and d and data not shown). These results are in line with previous evidence, suggesting that the antigen CD14, which is absent in untreated HL60 or expressed at low levels in the MonoMac-6 (Figures 2c–f), is a critical receptor involved in the monocyte responses to LPS^56^ Of note, the silencing of Ago2 protein reduces the D3- and D3 plus LPS-dependent induction of M-CSF and GM-CSF mRNAs in both the cell lines we tested (Figure 6a). Accordingly, the siAgo2 cells treated with D3 plus LPS released lower amounts of M-CSF and GM-CSF in the media relatively to the PGK cells (Figure 6b). In addition, Ago2-depleted HL60 cells also show, after the combined treatment, a decreased differentiation level, as assessed by morphological analysis and expression of the monocytic differentiation-related genes such as *MSE*, *MafB* and *M-CSFr* (Figures 6c and d).

Our observations indicate that Ago2 expression is required not only for the induction of monocytic differentiation but also to guarantee the cytokine production capability of monocytic cells.

## Discussion

MicroRNAs (miRNA) are emerging as constituents of evolutionarily highly conserved molecular pathways regulating cell fate decision in several developmental programs.^57^

Many efforts have been focused in these last years on the disclosure of the miRNAs involved in the establishment of myeloid differentiation and leukemogenesis. At the same time, with the identification of the members of Ago protein family as direct interaction partners of miRNAs within the effector complex RISC, a new level of regulation of miRNA activity is emerging. In this context, Ago2, the most abundant Ago protein in somatic cells specifically required for early mouse development, shows specific functions during miRNA processing and cell maturation.^58^ Here we present evidence that Ago2 protein expression is increased during monocyte differentiation of leukemic myeloid progenitors, whereas it is downregulated during granulocyte differentiation of human leukemic cell lines and freshly isolated blasts from acute promyelocytic leukemia patients. The silencing of Ago2 protein reduces vitamin D-dependent monocytic differentiation and supports RA-dependent granulocytic differentiation. On the contrary, recent results evidenced that the maintenance of Ago1 protein expression is necessary for the induction of RA-dependent granulocytic differentiation pathway.^26^ Together, these results suggest the selective contribution of different Ago family proteins to the establishment of alternative cell differentiation programs.

Here, we describe the requirement of Ago2 expression for a successful differentiation response of myeloid cells to 1,25-dihydroxyvitamin D_3_. Interestingly, Ago2-depleted cells fail to downregulate specific oncogenic miRNAs during monocytic differentiation. Ago2 protein thus seems to be directly responsible for the downmodulation of a subset of microRNAs during differentiation. This function probably relies on its ability to interact with transcription regulatory regions present on miRNA genes, modulating miRNA expression. We indeed obtained evidence that Ago2 localizes on miR-155HG and lncRNA-155HG regulatory regions where it is involved in the epigenetic regulation of their expression levels through the modulation of the chromatin conformation of the promoter region. Further studies will be required to elucidate whether miRNA processing and binding functions are necessary for Ago2-dependent epigenetic control at miRNA loci and to determine whether such novel factor may represent an additional molecular target for the establishment of leukemogenesis.

Overall, our findings are in agreement with and extend those of a recent study, demonstrating that the Dicer1-deficient granulocyte-macrophage progenitors (GMPs) are unable to mature toward monocytes, macrophages and myeloid DCs and that Dicer-deficiency leads to neutrophil dysplasia in mice.^59^ Furthermore, recent findings evidence the involvement of Ago2 protein phosphorylation in miRNA-mediated control of macrophage activation.^60^

Altogether these results highlight a central role for RISC proteins throughout the execution of the gene expression programs responsible for human myeloid cell fate determination.

## Materials and Methods

### Reagents

All-*trans*-retinoic acid (RA) and 1,25-dihydroxyvitamin D_3_ (D3) were purchased from Sigma-Aldrich (St. Louis, MO, USA) and utilized at a concentration of 1 *μ*M and 250 ng/ml, respectively, unless differently specified. The lipopolysaccharide (LPS) and cytosine-*β*-D-arabinofuranoside (ARA-C) were purchased from Sigma-Aldrich and utilized at a concentration of 1 *μ*g/ml and 1 *μ*M, respectively.

### Cell lines and cultures

SKNO-1, Kasumi-1, HL-60, NB4, ML-2, Me-1, THP-1, U937, HEL, K562 and Jurkat cell lines were maintained in RPMI 1640 medium supplemented with 1 × penicillin/streptomycin solution, 1 × L-glutamine and 10% Fetal Bovine Serum. MonoMac-6 cell line was maintained in 90% RPMI 1640+10% FBS+2 mM L-glutamine+non-essential amino acids+1 mM sodium pyruvate+10 *μ*g/ml human insulin. Human BM and PB mononuclear cells were obtained from informed newly diagnosed APL patients and maintained in RPMI 1640 medium supplemented with 1 × penicillin/streptomycin solution, 1 × L-glutamine and 10% fetal bovine serum. Cases were classified according to the French-American-British classification^5^ and showed an initial percentage of circulating blasts more than 90%.

### Cell proliferation/differentiation

Cell proliferation and differentiation were evaluated and quantified by (a) direct cell counting (trypan blue dye exclusion method) using a hemocytometer chamber; (b) conventional light-field microscopy morphological examination of Wright-Giemsa-stained cytospins; (c) NBT dye reduction assay (at least 500 morphologically intact cells for experimental condition were counted and corrected for viability, measured by trypan blue exclusion method); (d) direct immunofluorescence analysis of cell surface antigens using an Allophycocyanin (APC) anti-human CD11b (BD Biosciences, San Jose, CA, USA), PerCP-Cy5.5 anti-human CD14 (BD Biosciences) and PE-IgG1 isotype control (eBiosciences Inc., San Diego, CA, USA) for the detection of the CD11b-CD14 co-expression as a marker of monocytic differentiation, as previously described.^61^ A minimum of 10 000 events was collected for each sample with a flow cytometer (CyAN ADP DAKO) by using Summit 4.3 software (Beckman Coulter, Fullerton, CA, USA) for data acquisition and analysis. Cytochemical staining of *α*-naphthyl acetate esterase (ANAE) and ANAE inhibited by NaF was performed according to the manufacturer's instructions (Sigma-Aldrich). For cell cycle analysis, 2 × 10^5^ cells were resuspended in 50% FCS, fixed in 70% ethanol for 24 h, incubated with 50 Ag/ml propidium iodide (Sigma-Aldrich) and 50 units/ml Dnase-free RNase A (Sigma-Aldrich) and analyzed after 3 h (10 000 events) using an Epics XL Cytometer (Beckman Coulter).

### Lentiviral shRNA expression constructs

The shRNA expression cassette for Ago2 silencing was generated by cloning the following sequence: 5′-GAT CCA CTA GCT GTG AAT CTT CTG TTC AAG AGA CAG AAG ATT CAC AGC TAG TTT TTT TGG AAA-3′(forward) and 5′-AGC TTT TCC AAA AAA ACT AGC TGT GAA TCT TCT GTC TCT TGA ACA GAA GAT TCA CAG CTA GTG-3′ (reverse) into the *Bgl*II-*Hin*dIII-digested pSR-GFP/Neo plasmid; the *Eco*RI-*Xho*I fragment from the last (pSR-GFP/Neo plasmid) was then subcloned into the *Eco*RV site of the lentiviral vector pRRLcPPT.hPGK.EGFP.WP. Infective particles were produced and utilized as previously described.^62^

### RNA extraction and analysis

Total RNA from cells was extracted using the TRIzol RNA isolation system (Invitrogen, Carlsbad, CA, USA) according to the manufacturer's instructions and reverse transcribed with random primers and SuperScript II reverse transcriptase (Invitrogen). The cDNA was used for quantitative real-time PCR (qRT-PCR) experiments carried out in an ABI PRISM 7000 Sequence Detection System (Applied Biosystems, Carlsbad, CA, USA). Taqman oligonucleotides (Assay-on-Demand) for argonaute-2 (Ago2) Hs00293044_m1, glyceraldehyde-3-phosphatedehydrogenase (GAPDH) Hs99999905_m1, monocyte/macrophage serine esterase-1 (MSE or CES-1) Hs00275607_m1, v-maf muscoloaponeurotic fibrosarcoma oncogene homolog B (MafB) Hs00534343_s1, colony-stimulating factor 1 receptor (CSF1R or M-CSFr) Hs00234622_m1, trasglutaminase type-II (TGase-II) Hs00190278_m1, granulocyte-macrophage colony-stimulating factor 2 (CSF2 or GM-CSF) Hs99999044_m1 and macrophages colony-stimulating factor 2 (CSF1 or M-CSF) assay Hs00174164_m1 were from Applied Biosystems. ΔΔCt values were normalized with those obtained from the amplification of GAPDH. The SYBR Green dye detection method for the retinoic acid receptor beta (RAR*β*) amplification was performed as previously described.^37^ The quantification of Hsa-miR-17-5p, Hsa-miR-20a, Hsa-miR-106a, Hsa-miR-125b, Hsa-miR-155, Hsa-miR-26a and Hsa-miR-155HG (Hs01374570_m1) was carried out in an ABI PRISM 7000 Sequence Detection System (Applied Biosystems) with TaqMan MicroRNA Assay (Applied Biosystems), whereas the quantification of the lncRNA-155HG was performed in the same experimental condition using the SYBR Green dye detection method with the following oligo sequences: lncRNA-155HG_for 5′-ATTGCACAGC AATGTCCTGATGGC-3′ and lncRNA-155HG_rev 5′-ACACTGGAGAGACAAAGGTGAGCA-3′. ΔΔCt values were normalized with those obtained from the amplification of the endogenous U6B snRNA (Applied Biosystems). All reactions were performed in triplicate.

### Western blot analysis

Thirty micro grams of whole-cell extract was separated by 4–12% SDS–PAGE (Invitrogen) and electroblotted to nitrocellulose membrane (Protran, Whatman S&S, Maidstone, UK). Whole-cell extract from primary APL mononuclear cells (3 × 10^6^) were obtained using 30 *μ*l of CelLytic MT reagent (C-3228 Sigma-Aldrich), added with PhosSTOP and Complete Mini EDTA-free Protease Inhibitor (F. Hoffmann-La Roche AG Konzern-Hauptsitz, Basel, Switzerland). Immunoblots were incubated with the following rabbit polyclonal antibodies: anti-Ago2 (#2897; Cell Signaling Technology, Inc., Danvers, MA, USA) anti-AML1b (#4334, Cell Signaling Technology, Inc.), anti-VDR (ab3508; Abcam plc, Cambridge, UK), anti-c-Jun (sc-45, Santa Cruz Biotechnology, Inc., Santa Cruz, CA, USA) and anti-C/EBP*β* (sc-150, Santa Cruz Biotechnology, Inc.). The anti-*α*-tubulin mouse monoclonal IgG (Sigma-Aldrich) was used to normalize the amount of the samples analyzed. The immunoreactivity was determined using the enhanced chemiluminescence (ECL) method (Amersham Biosciences, Fairfield, CT, USA). Densitometric measurements were obtained by the Quantity One software (Bio-Rad Laboratories, Hercules, CA, USA).

### Chromatin immunoprecipitation assay

ChIP assays were performed as described^37^ using antibodies anti-acetyl-Histone-H4 (H4Ac) (Merk Millipore, Billerica, MA, USA; cat#06–866) and anti-Ago2 (Merk Millipore, cat#03–110). Genomic regions on miR-155HG and lncRNA-155HG were amplified using primers designed by the Primer 3 software (http://www-genome.wi.mit.edu/genome_software/other/primer3.html). qRT-PCR was performed in triplicate samples using the SYBR green dye detection method and the following primers: primer 1 for miR-155HG_for 5′-ATGTCCTGGAGATGGGAGTG-3′ and miR-155HG_rev 5′-CCCTACCTCATCACCCTTCA-3′ primer 2 for lncRNA-155HG_for 5′-TTCTCATCT GCCCTGAAACA-3′ and lncRNA-155HG_rev 5′-TTGCTGAGAGCCTTGGACTT-3′. Values obtained for each immunoprecipitated sample were quantified *versus* the respective input and calculated following the 2−ΔCT method.

### ELISA assay

The cells were plated and treated with vitamin D 2.5 ng/ml plus LPS 1 *μ*g/ml. After 96 h, the medium was collected and centrifuged at 1200 r.p.m. for 10′ to remove cells' contamination. To analyze the amount of M-CSF or GM-CSF released in the medium from HL60 and Monomac-6 cells after treatment, we performed an ELISA (Enzyme-Linked Immunosorbent Assay) using the RayBio Human M-CSF/GM-CSF ELISA Kit (ELH-MCSF-001, ELH-GMCSF-001 RayBiotech, Inc., Norcross, GA, USA). The assay was performed according to the manufacturer's instructions, and OD_450_ readings were obtained with the plate reader Multiskan FC, Thermo Fisher Scientific (Waltham, MA, USA).

## Abbreviations

AML: acute myeloid leukemia
RA: retinoic acid
D3: 1,25-dihydroxyvitamin D_3_
miRNA: microRNA
Ago: argonaute
RISC: RNA-induced silencing complex
lncRNA: long noncoding RNA
UTR: untranslated region
ARA-C: arabinofuranosyl Cytidine
HDAC: histone deacetylases
VDR: 1,25-dihydroxyvitamin D_3_ receptor
LPS: lipopolysaccharide
GM-CSF: granulocyte-macrophage colony-stimulating factors
M-CSF: macrophage colony-stimulating factors
NBT: nitroblue tetrazolium
ANAE: acid alpha-naphthyl acetate esterase
