# Supplementary figures and images for: Argonaute 2 sustains the gene expression program driving human monocytic differentiation of acute myeloid leukemia cells
Source: Cell Death Dis. 2013 Nov 21;4(11):e926–. doi: 10.1038/cddis.2013.452 (PMC3847328; doi:10.1038/cddis.2013.452)

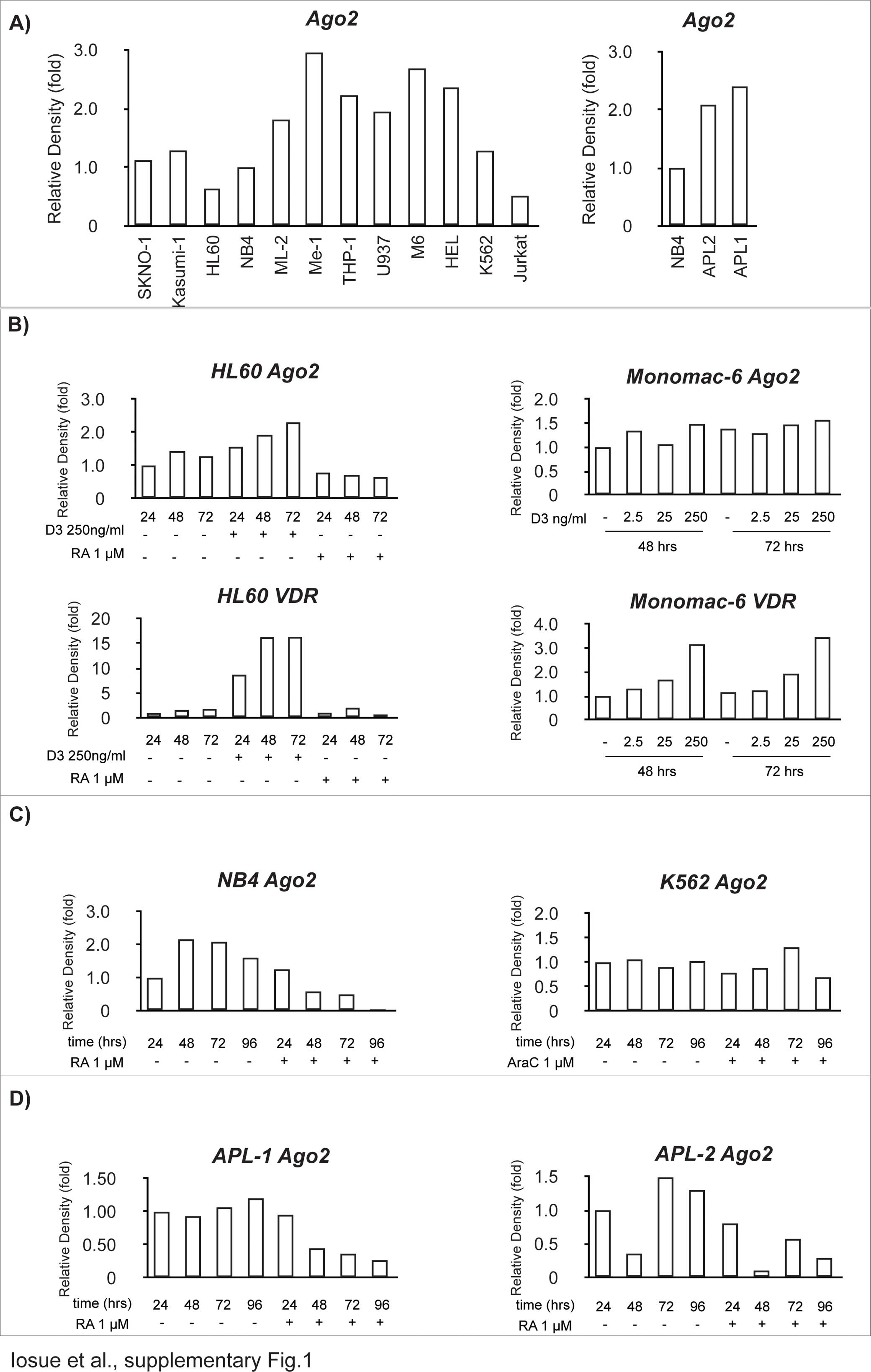

Supplement: Supplementary Figure 1 [file cddis2013452x1.tif]

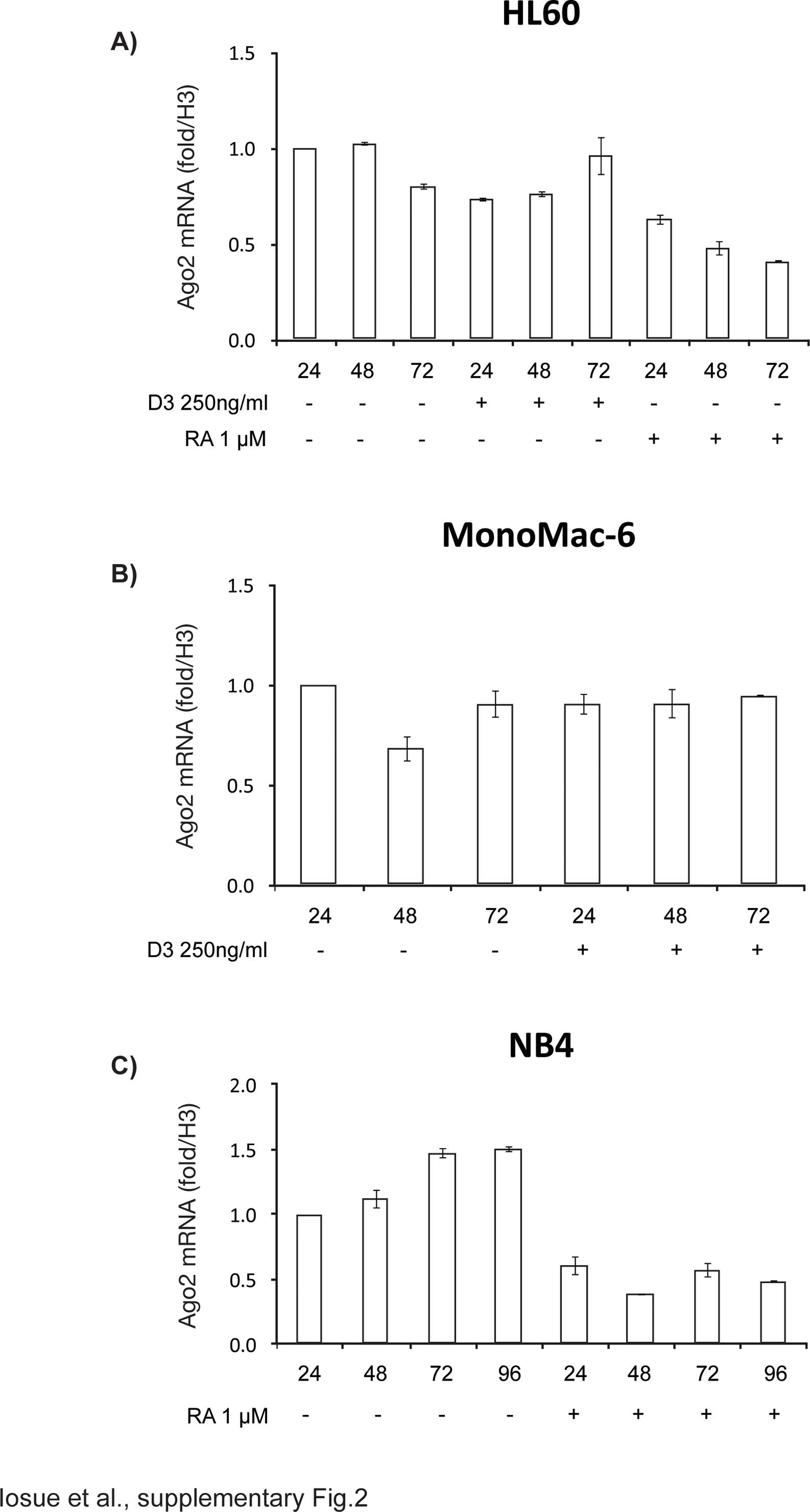

Supplement: Supplementary Figure 2 [file cddis2013452x2.tif]
