# Supplementary material for: Argonaute 2 sustains the gene expression program driving human monocytic differentiation of acute myeloid leukemia cells
Source: Cell Death Dis. 2013 Nov 21;4(11):e926–. doi: 10.1038/cddis.2013.452 (PMC3847328; doi:10.1038/cddis.2013.452)
Supplement: Supplementary Information [file cddis2013452x3.doc]

Legend to Supplementary Figures

S.1

Densitometric analysis of the western blots in Fig.1 was performed with the Quantity One Software (BioRad Laboratories), using tubulin for normalization. Data are reported as relative density.

S.2

Ago2 mRNA expression was evaluated by qRT-PCR in HL60, MonoMac-6 and NB4 cells treated or not with D3 or RA as indicated. PCR data were analyzed by the ΔΔCt method (n=3 ± SEM) using GAPDH for normalization.
